# The emergence and shift in seasonality of Lyme borreliosis in Northern Europe

**DOI:** 10.1098/rspb.2022.2420

**Published:** 2023-02-22

**Authors:** Asena Goren, Hildegunn Viljugrein, Inger Maren Rivrud, Solveig Jore, Haakon Bakka, Yngvild Vindenes, Atle Mysterud

**Affiliations:** ^1^ Centre for Ecological and Evolutionary Synthesis (CEES), Department of Biosciences, University of Oslo, PO Box 1066 Blindern, Oslo NO-0316, Norway; ^2^ Norwegian Veterinary Institute, PO Box 64, NO-1431 Ås, Norway; ^3^ Norwegian Institute for Nature Research (NINA), Sognsveien 68, NO-0855 Oslo, Norway; ^4^ Zoonotic, Food and Waterborne Infections, The Norwegian Public Health Institute, PO Box 4404 Nydalen, NO-0403 Oslo, Norway; ^5^ Norwegian Institute for Nature Research (NINA), PO Box 5685 Sluppen, NO-7485 Trondheim, Norway

**Keywords:** seasonality, vector-borne zoonoses, disease ecology, Lyme borreliosis, Lyme disease, climate change

## Abstract

Climate change has had a major impact on seasonal weather patterns, resulting in marked phenological changes in a wide range of taxa. However, empirical studies of how changes in seasonality impact the emergence and seasonal dynamics of vector-borne diseases have been limited. Lyme borreliosis, a bacterial infection spread by hard-bodied ticks, is the most common vector-borne disease in the northern hemisphere and has been rapidly increasing in both incidence and geographical distribution in many regions of Europe and North America. By analysis of long-term surveillance data (1995–2019) from across Norway (latitude 57°58′–71°08′ N), we demonstrate a marked change in the within-year timing of Lyme borreliosis cases accompanying an increase in the annual number of cases. The seasonal peak in cases is now six weeks earlier than 25 years ago, exceeding seasonal shifts in plant phenology and previous model predictions. The seasonal shift occurred predominantly in the first 10 years of the study period. The concurrent upsurgence in case number and shift in case timing indicate a major change in the Lyme borreliosis disease system over recent decades. This study highlights the potential for climate change to shape the seasonal dynamics of vector-borne disease systems.

## 1. Introduction

Emergence and range expansion of vector-borne and other infectious diseases are expected to accompany the many threats of the climate crisis [1,2]. Climate warming and environmental changes are suspected to have already led to both geographical range expansion of many vector-borne diseases and increased incidence in regions where diseases are already established [3–5]. Ecosystems at northern latitudes are experiencing above-average climate warming, which can create more favourable conditions for arthropod disease vectors and thus increase disease hazard [6,7]. In addition to warming, climate change has modified the seasonal structure in northern latitudes, introducing shorter winters, an earlier onset of spring and a longer growing season [8]. Phenological changes in the activity patterns of organisms have become one of the most notable effects of climate change in temperate regions and can be observed across taxa, from changes in the onset of plant growth, to reproductive timing in birds and mammals [9,10].

The speed at which different species respond to climate warming varies, and asynchronous responses by interacting species can create phenological mismatches [11–14]. Vector-borne zoonoses are maintained by inherently complex ecological networks of hosts, vectors and pathogens [2]. Phenological overlaps between interacting components are key determinants of disease dynamics, and climate change-induced asynchronies are likely to alter disease trends. Empirical evidence demonstrating linkages between climate change, seasonality and disease outcomes has been limited [15,16]. Analysis of consistent, long-term surveillance data has been recognized as a critical step in developing an understanding of the ecological and climatic drivers of disease risk [3,4,15]. Explicit consideration of case seasonality in surveillance data can improve the detection of long-term changes in disease trends and is important for identifying potential effects of climate change on disease dynamics [17–21].

Lyme borreliosis, or Lyme disease, is a zoonotic infection caused by certain genospecies of the *Borrelia burgdorferi sensu lato* (sl) complex that are transmitted by tick vectors in the genus *Ixodes* [22]. The ixodid tick vectors are highly generalist hematophages that feed on a wide range of vertebrates including small and large mammals, birds and reptiles. A blood meal is required for the tick to develop between life stages, from larvae to nymphs and then adults, and for adult females to lay eggs [23]. *B. burgdorferi* sl is acquired by larval and nymphal ticks during feeding on infected hosts, and then transmitted during subsequent feedings [24]. The different pathogenic *B. burgdorferi* sl genospecies are associated with different vertebrate groups. In Europe, *Borrelia afzelii* is found in small mammals and *B. garinii* in birds [25]. In North America, *B. burgdorferi sensu stricto* is the main pathogenic genospecies and is found in both mammals and birds [26]. Hence, the circulation in the ecosystem of pathogens causing Lyme borreliosis differs markedly between the continents.

Lyme borreliosis is the most common vector-borne disease across temperate regions of the northern hemisphere [27–30]. Over recent decades, there has been an increase in both the number of Lyme borreliosis cases and the geographical distribution range, with emergence particularly impacting northern latitudes and high-elevation regions in North America and Europe [27,31–34]. Several studies have investigated spatial disease trends and the environmental factors that influence regional disease risk [33–37]. For temporal disease trends, empirical exploration of seasonality change is restricted to cases in the USA [38–40]. However, as the European and North American disease systems differ fundamentally due to contrasting hosts, pathogens and vectors, it is necessary to consider these systems independently [41]. For the European disease system, changes in Lyme borreliosis seasonality have only been predicted by a mechanistic model based on data from Scotland [42]. Our study is the first in Europe to use surveillance data to explore changes in the seasonality of Lyme borreliosis cases.

The goal of this study is to quantify changes in both the incidence and seasonal timing of Lyme borreliosis cases at the expanding northern distribution range in Europe. Lyme borreliosis surveillance data have been consistently reported in Norway under uniform criteria since 1995, presenting an excellent data source for this undertaking. Furthermore, Norway comprises distinct ecoregions with differences in climate and host composition, which allows for a unique opportunity to compare disease seasonality in ecologically distinct areas unified under a single-surveillance umbrella. Changes in plant phenology, described by spring greening measured from satellite data using the Normalized Difference Vegetation Index (NDVI), are used as a yardstick for interpreting the magnitude of phenological responses to climate change in the study area [9].

## 2. Methods

### (a) Study area

**Figure 1.**
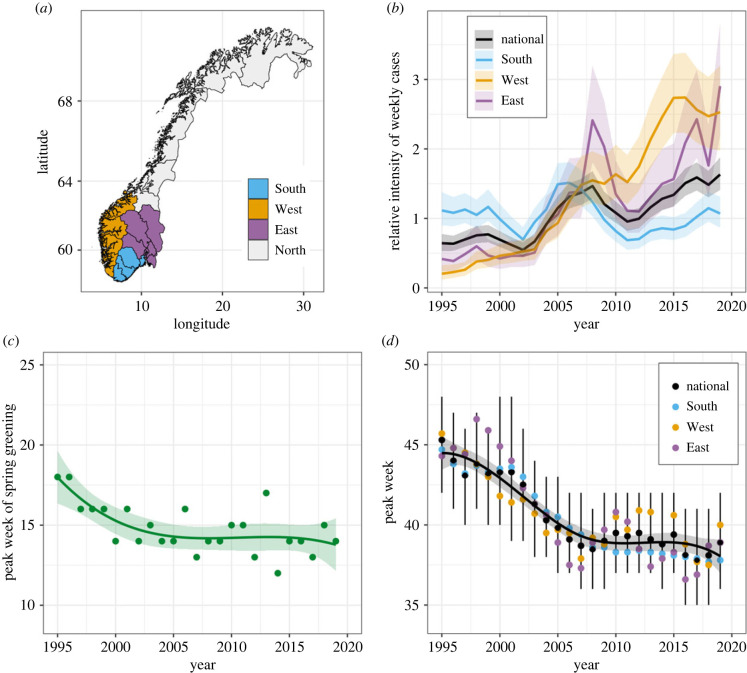
Key changes in the seasonal and long-term trends of Lyme borreliosis. (*a*) Map of Norway showing the regional aggregations used in this, and prior, studies [25,34]. The statistical models are fitted to national data, as well as to the South, West and East regions independently. (*b*) The annual component from the main model for Lyme borreliosis cases fitted to the national data (black) and to regional data for the South (blue), West (yellow) and East (purple). The trends show relative changes in average weekly case totals predicted for each year (relative case intensity). Because the intercept is not included, the trends are not comparable on an absolute scale. (*c*) The shift in the week of peak spring vegetation greening, measured by NDVI. The points represent the week in which peak greening was observed. The trendline is fitted from a linear model with a basis spline for year, with three degrees of freedom. (*d*) Predicted peak weeks for Lyme borreliosis cases from the main national model (black) and the regional models fitted to the South, West and East (blue, yellow and purple). Black points represent the annual peaks from the national model, and the corresponding black vertical lines show the 95% credible intervals, quantified by repeated sampling from the posterior distribution. The black curve with a shaded confidence interval is a basis spline with four degrees of freedom fitted to the predicted national peaks.

Norway's Lyme borreliosis surveillance data include cases reported from the entire country, spanning a latitudinal range of 57°58′–71°08′ N [34,43]. For this analysis, cases reported at the municipality scale were grouped into four biogeographical regions, North, South, East and West (figure 1*a*), following designations from prior studies [25,34]. The four regions represent contrasting ecosystems with marked differences in topography and climate. The West region is separated from the East by a mountain range and experiences a temperate maritime climate, in contrast with the more continental climate of the East. The region South is mild and humid [44]. Forest and species composition also differ between regions [45]. Large mammal communities in particular are different between regions, which has especial importance for the vector life cycle [46]. The region West is dominated by red deer (*Cervus elaphus*), while roe deer (*Capreolus capreolus*) and moose (*Alces alces*) are most prominent in the regions East and South. Generally, the same small mammal and avian host species occur across the regions studied, though quantitative evidence of differences in abundances and host importance between regions remains limited [24,47].

Documented effects of climate change have been recorded across Norway over the study period [48]. For the reference period of 1979–2008, the annual mean temperature for mainland Norway has increased by 0.5–0.6°C, with winter temperatures increasing by about 1°C. The growing season, defined by the numbers of days with a mean temperature above 5°C, has increased by 1–2 weeks nationally, with the greatest increase in the Western coastal regions. Annual precipitation has increased in all regions, on average by 3% per decade, with the Western region most impacted by increased precipitation. The precipitation has primarily increased in the spring and decreased in the autumn [48]. The streamflow during the spring has also increased due to earlier snowmelt and earlier timing of snowmelt-driven flooding events [48,49]. The snow season has become shorter in most parts of mainland Norway, with reduced annual snow depth and fewer days of snow cover [48]. Advancement in spring plant phenology in response to climate change has been documented [50,51].

### (b) Lyme borreliosis surveillance data

Lyme borreliosis surveillance data has been collected by the Norwegian Surveillance System for Communicable Diseases (MSIS) since 1991, when it became mandatory for care providers to report cases of positive diagnosis [52]. MSIS is curated and administrated by the Norwegian Institute of Public Health. The study period used for this analysis is from 1995 to 2019 because consistent criteria for reporting cases of disseminated Lyme borreliosis have been maintained over this time. A detailed account of reporting criteria has been reported elsewhere [25,52]. The only significant documented change in diagnostics over the study period is the standardization of spinal fluid testing protocols for children since 2011 [25]. Because of this change in testing protocol and to reduce bias from health system effects, case reports used for this study were restricted to patients over age 19. Cases were spatially localized to the region in which the tick bite occurred when these data were available (*ca* 50% of cases), otherwise the municipality in which the person resides was used. If the location of bite was reported as outside of Norway these cases were not included in the study. Case timing is based on the date on which the patient went for diagnostic testing, which is available for every case. This date will typically occur several weeks after the tick bite, allowing time for disseminated disease symptoms to have manifested [53].

### (c) Normalized difference vegetation index

Changes in the seasonality of plant development were used as a yardstick to contextualize the magnitude of temporal changes in the seasonality of Lyme borreliosis cases. Plant development was characterized using remote sensing NDVI satellite data to determine peak spring greening (when the rate of plant green-up is fastest) each year from 1995 to 2019. NDVI data are a widely used indicator of ecological responses to environmental change [54]. Changes in plant phenology are not expected to affect the timing of Lyme borreliosis cases directly, but are a useful point of reference for exploring downstream effects of climate change.

NDVI images are produced from satellite instrumentation that has been available from various sources since 1981 [55]. Moderate Resolution Imaging Spectroradiometer (MODIS) data at 250 m resolution have been available since 2000 and were downloaded from NASA Earthdata (https://urs.earthdata.nasa.gov/home) using the ‘MODIStsp’ package in R [56]. For the first 5 years of the study period (1995–2000), NDVI data from the Global Inventory Modelling and Mapping Studies (GIMMS) at the 8 km resolution scale were downloaded using the ‘gimms’ package in R [57]. MODIS and GIMMS data have been shown to be highly correlated and suitable for making continuous time series [55]. The high correlation (*r* > 0.9) was confirmed for this study by checking the years 2000–2005 in which data from both sources were available.

NDVI data were processed for areas below 200 m above sea level in the West, East and South regions combined, to estimate a yardstick that is on the national scale and relevant to areas in which Lyme borreliosis is most common. The MODIS NDVI images are collected once every 16 days, and the GIMMS NDVI images every 15/16 days. All images were processed following the procedures in Bischof *et al*. [58] and Rivrud *et al*. [59,60]. For each image pixel, the NDVI over the study period was scaled (0–1) and a double logistic regression curve was fitted annually following Bischof *et al*. [58] and Rivrud *et al*. [59,60] to estimate a continuous time series from which derivatives, e.g. rate of change in green-up, start of spring, end of spring, and more, can be calculated [58,60]. Further details on the processing and modelling of NDVI data can be found elsewhere [58–60].

The day on which the rate of increase in greenness was at the maximum was used to determine the date of peak spring greening, and the corresponding week number was then extracted to make the data comparable to the Lyme borreliosis case data. A linear regression using a basis spline with three degrees of freedom was applied to visualize the change in peak spring greening over the study period. The basis spline with three degrees of freedom was selected for having the lowest Akaike information criterion (AIC) in comparison with a linear model and more flexible splines (see electronic supplementary material).

### (d) Statistical analysis

The statistical software R v.4.2.2 was used for all statistical analyses [61]. The package INLA (http://www.r-inla.org) was used to fit all models. INLA uses a method of Integrated Nested Laplace Approximation to rapidly fit Bayesian models [62]. The R script (reproducing all the results and figures) and further details on the statistical analyses are available in the electronic supplementary material.

The number of Lyme borreliosis cases per week was modelled with a generalized linear mixed model (GLMM) including a flexible seasonal component that allows for the separation of a yearly trend and a seasonal trend. The number of cases *y_ij_* in week *i* (from 1 to 52) and year *j* were assumed to follow a Poisson distribution,

2.1 $$y_{ij}\;∼\;\mathrm{Poisson}(\lambda_{ij}),$$

where *λ_ij_* is the expected number of cases according to the model. As is standard for Poisson GLMMs, we used a logarithmic link function (i.e. ln(*λ_ij_*) is a linear predictor). The model formula was specified as follows:

2.2 $$\ln(\lambda_{ij})=\beta_{0}+\ln(N_{j})+Y_{j}+W_{ij}+\;\varepsilon_{ij},$$

where *β*_0_ is the intercept, *Y_j_* is the year effect (modelled as a first-order random walk), *W_ij_* is the seasonal effect (specified below), ln(*N_j_*) is the population offset and *ε_ij_* is a Gaussian random effect used to account for overdispersion [63].

The population offset ln(*N_j_*) is the logarithm of the total adult population *N_j_* in the region. This was included to account for any changes in the number of observed cases due to changes in population size. Including a population offset makes the log-linear model equivalent to modelling the log of the expected number of cases relative to the population size (i.e. ln(*λ_ij_*/*N_j_*)). Population size data were obtained on 25 January 2022 from Statistics Norway, Population Count (https://www.ssb.no/en/befolkning/folketall).

The main advantage of this model is its ability to separate the yearly (*Y_j_*) and seasonal (*W_ij_*) components, so that changes in seasonality can be isolated from long-term disease trends. The modelling approach for the seasonal component was inspired by an analysis of monthly registered cases of mumps in New York City by Ruiz-Cárdenas *et al*. in 2012 [64]. The long-term trend (*Y_j_*) is modelled as a first-order random walk (prior specified in the electronic supplementary material). The seasonal component is based on a periodic function, where the effect of week number *i* in year *j* is given by

2.3 $$W_{ij}=\beta_{ij}\sin\left(\frac{2\pi }{52}\right)+\;\gamma_{ij}\cos\left(\frac{2\pi }{52}\right),$$

where *β_ij_* and *γ_ij_* are each modelled with a first-order random walk to make the seasonal effect of sequential weeks highly correlated, and the constant factor 2π/52 defines the period in weeks within year. This seasonal effect is smooth across the study period because the parameters *β_ij_* and *γ_ij_* vary slowly.

Using trigonometric identities, equation (2.3) above can be rewritten as follows:

2.4 $$W_{ij}=A_{ij}\sin\left(\frac{2\pi }{52}+p_{ij}\right),$$

where *A_ij_* represents the amplitude (determined by $A_{ij}^{2}=$ $\beta_{ij}^{2}+\gamma_{ij}^{2}$), and *p_ij_* represents the phase shift of the sinusoidal function (determined by tan(*p_ij_*) = *β_ij_*/*γ_ij_*). Importantly, the phase shift in equation (2.4) uniquely identifies a peak week of each year during which the number of Lyme borreliosis cases is at the maximum predicted by the model. Thus, changes in case seasonality over the study period can be quantified by extracting the peak week for each year from the model. In total, this results in a flexible seasonal component that can capture changes in seasonality over time. Credible intervals (95%) for the annual seasonal peaks were computed from 1000 samples from the posterior model. Long-term trends in annual seasonal peaks were visualized by fitting a basis spline with four degrees of freedom using the ‘ggplot2’ package in tidyverse [65].

### (e) Regional analysis

To compare regional differences in seasonality, the model described above was fit to the South, West and East regions separately, as well as to national data that includes all regions. The North region contained only few cases (approx. 5% of total cases) and was not analysed separately. Fitting the models for each region separately can reveal any differences between the regions in the seasonality and long-term trends of cases and indicate if changes in the geographical distribution of cases could underlie national shifts in case timing. Regional models accounted for local adult population offsets, using data obtained from Statistics Norway.

### (f) Model selection and alternative models

To determine whether case seasonality changed over the study period, the main model described above was compared with two alternative models with fixed seasonality across years. The alternative models only differed from the main model in the construction of the seasonal component. All models were fitted using the national dataset for this comparison.

The first alternative model removed the flexibility of the seasonal component by fixing the amplitude *A_ij_* and the phase *p_ij_* in the periodic function (equation (2.4)) so that they are the same each year. An additional alternative model with an improved description of a fixed seasonality was modelled using a cyclic random walk to fit a flexible spline with one knot per week [62,66]. This improved alternative model has a closely tailored seasonal component compared to the coarser sinusoidal seasonal model. Comparing these models allowed us to determine whether a closely fit but fixed seasonal trend describes the data better than a more coarsely fit but flexible seasonal trend that changes over time.

Comparison of the models was done through cross-validation (see electronic supplementary material). A randomly selected 10% of the data were removed before each model was refitted, and model performance was compared across 10 repetitions. Prediction quality was scored by root-mean-square error (RMSE), mean absolute error (MAE) and the negative log-likelihood (NLL) [67]. Additionally, the deviance information criterion (DIC) score, as given by INLA, was used as a metric for model comparison [68].

## 3. Results

**Figure 2.**
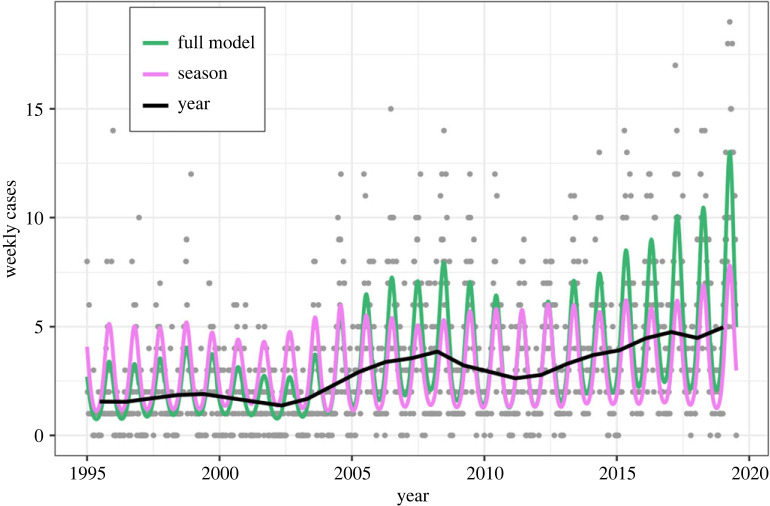
Weekly case numbers over the study period (grey points), together with model predictions (green) for the main national model. The yearly trend is shown in black (seasonal effect estimates set to zero), and the seasonal trend is shown in magenta (yearly effect estimates set to zero).

The main model with a flexible seasonal trend that changes across years demonstrated good fit throughout the study period (figure 2; electronic supplementary material, figure S6) and performed well in cross-validation assessments (electronic supplementary material, figure S16). The main model outperformed both alternative models with fixed seasonality on all cross-validation metrics and using DIC (table 1), indicating that there has been a shift in Lyme borreliosis case seasonality over the study period (1995–2019). With the main model, we were able to separately quantify year-to-year changes in the number of cases and changes in within-year seasonality. The main model's parameters specifying the random effect distributions are reported in the electronic supplementary material, table S1. The seasonal component accounted for a larger amount of the total variance than the year component, highlighting the importance of including the seasonal trend (electronic supplementary material, table S2). There was no remaining temporal autocorrelation in the residuals (electronic supplementary material, figure S13). The unimodal seasonality employed by the main model is supported by the cross-validation and through analysis of the residuals, which show that the seasonality component of the main model is descriptive across weeks within the year (electronic supplementary material, figure S14). Thus, our findings support that the seasonality of Lyme borreliosis cases in Norway is characterized by a single main peak.

**Table 1.** Model performance metrics from cross-validation and DIC for comparing different candidate structures for the seasonality component. The main model includes a flexible seasonal component, while the two alternative models include a fixed seasonal component (see main text and electronic supplementary material). The metrics used were RMSE, MAE, NLL and DIC.

model	RMSE	MAE	NLL	DIC
main model	2.08	1.53	2560.5	5065.5
fixed cyclic random walk	2.12	1.56	2589.6	5096.0
fixed sinusoidal	2.13	1.57	2599.2	5104.2

There was a clear national trend of increasing relative case intensity over time (reflecting the year component of the main model), with strong regional differences (figure 1*b*). While nationally the average weekly cases have more than tripled over the study period, there has only been a small increase in the region South (figure 1*b*). This is in contrast with the West, where in 2019 there were more than 10 times as many cases than at the start of the study period. The region South reported the majority of raw case counts in Norway every year until 2010, when the West surpassed it in the proportion of total cases reported annually. However, throughout the study period, the South maintained the highest annual incidence (number of cases per 100 000 adults) due to the much smaller population size relative to the other regions (electronic supplementary material, figure S9). Statistics describing trends in the raw data can be found in the electronic supplementary material.

There was a distinct change in the seasonal timing of Lyme borreliosis cases, characterized by an earlier shift in the week in which case numbers peaked (figure 1*d*). The extent of the seasonal shift was around six weeks over the 25-year study period, averaging to a rate of change of around 0.2 weeks per year. In the mid-1990s, the seasonal peak was typically in late October, while in 2018 it was in early September. Most of the seasonal shift occurred in the first 10 years of the study period, after which the peaks have been more stable. This is illustrated in figure 1*d*, where the trend line fitted to annual peaks is a basis spline with four degrees of freedom, which had a lower AIC than a linear model (difference of 7.8). No regional differences in the seasonal shift over the study period were observed. Each regional model exhibited the same overall pattern as the national model, and all regional peaks were within the credible intervals of the national model (electronic supplementary material, figures S11 and S12).

The shift in case seasonality outpaced the NDVI yardstick, which showed a shift towards an earlier peak spring greening of around three weeks over the study period (figure 1*c*). As with case timing, no regional differences in the NDVI trend were observed. Although the shift in NDVI was less pronounced than the shift in Lyme borreliosis cases, both had a similar overall trend with a rapid shift in the first 10 years and then a stabilization for the rest of the study period.

## 4. Discussion

Climate change is expected to impact the dynamics and geographical extent of vector-borne diseases, but simultaneous disease emergence and changing seasonality have rarely been quantified. Lyme borreliosis exhibits a highly seasonal incidence pattern and high sensitivity to environmental conditions, making it an interesting case study for exploring changes in seasonality. By applying a statistical model framework that explicitly accounts for seasonality in long-term surveillance data, we provide the first empirical evidence supporting a marked shift in Lyme borreliosis seasonality accompanying disease emergence and major climatic changes in northern latitudes of Europe.

Alteration of seasonality is a critical pathway by which climate change can affect ecosystem dynamics [69,70]. Changes in the seasonality of Lyme borreliosis cases were consistent between ecoregions of Norway (figure 1*d*), despite major regional differences in the species composition of large vertebrate hosts. All regions share the same tick vector, indicating that changes in vector phenology are likely an important driver of the observed patterns of Lyme borreliosis cases. Lyme borreliosis infections have been shown to peak in synchrony (with a lag) with high activity levels of nymphal ticks [38,39,42]. Ticks have the capacity for rapid modification of their questing activity and stage durations on the individual scale, yielding high plasticity in their life-history responses to environmental drivers [23,71–73]. The findings in this study support the intuitive effect of warmer springs shifting the Lyme borreliosis season earlier.

It has been hypothesized that warmer spring weather could potentially lead to increased nymphal tick activity in autumn due to a shortened development cycle leading to a second annual peak of the nymph stage [23,38,74,75]. A mechanistic model based on data from Scotland predicted increased incidence rates and a lengthening of the Lyme borreliosis season, but small changes in seasonality that varied between regions, with some regions having an earlier shift and other regions having a later shift in seasonality [42]. Our empirical analysis found only a consistent earlier shift in Lyme borreliosis cases in Norway, across all ecoregions. Both unimodal and bimodal distributions of tick questing activity levels have been observed in Europe, typically with a strong spring activity peak and, in some regions, a second, smaller autumn activity peak [76,77]. In Norway, tick activity data are limited to one study site in the Western region, where it was found that tick questing levels peaked in early summer (May–June), and only in some years, there was also a small activity peak in early autumn [64]. We found no evidence for a later secondary peak of Lyme borreliosis cases (electronic supplementary material, figures S1 and S13). This is consistent with findings in Denmark, where tick activity levels are reported to have a bimodal pattern while human Lyme borreliosis cases have a unimodal distribution [78–81]. These findings suggest that the seasonal pattern of Lyme borreliosis cases is driven by processes other than tick activity levels alone. In Norway, pathogen prevalence in small mammal hosts has been found to be consistently higher in spring than in fall, while pathogen prevalence in questing ticks is seasonally variable across years [24,34]. Human activity patterns may also lead to variable exposure across seasons.

The shift in Lyme borreliosis seasonality observed in this study far exceeds the magnitude of change observed in the USA [38,40]. An empirical study of Lyme borreliosis cases over a similar time period (1992–2007) across 12 U.S. states found that warmer southern states had an earlier seasonal onset than colder northern states [38]. However, there was high inter-annual variability in timing of cases and no consistent shift in seasonality [38]. The value of using a yardstick has been highlighted when comparing quantitative estimates of phenology [9]. For this study, NDVI was selected because it is a well-developed index for the onset of plant growth [54]. The seasonal shift in Lyme borreliosis cases documented here paralleled the shift in the onset of plant growth but with a larger magnitude of shift (figure 1*c,d*). The difference in magnitude of shift between peak spring greening and Lyme borreliosis case timing highlights that there are likely differences in the climatic drivers of these two manifestations of climate change. Empirical evidence suggests that several species of arthropods are highly sensitive to climate change and can show more rapid phenological shifts than plants and vertebrates [9,82]. It remains a gap in current understanding of tick biology to what extent tick stage durations are controlled by photoperiod or temperature [83,84]. The rapid seasonal shift observed in this study suggests that photoperiod has a limited effect on tick emergence from winter diapause, and that climatic drivers, such as increasingly warm spring temperatures and shifts in spring moisture levels and snow melt, primarily drive the tick life cycle.

Alternative or additional explanations for the rapid shift in seasonality of Lyme disease cases other than changes in tick vector phenology cannot be excluded. Phenological changes have also been documented in many host species linked to the Lyme borreliosis disease system, such as onset of the deer calving season [85,86] and the timing of migration and reproduction of birds [87]. Migratory birds play an important role in the Lyme borreliosis disease system [47,88], but whether changes to avian host phenology can drive the marked changes in Lyme borreliosis disease seasonality remains unclear. Changes to the healthcare system could underlie unexpected epidemiological patterns observed over long study periods. It is possible that improvements to health technologies and increased disease awareness among physicians and the public may have contributed to some of the seasonality shift observed over the study period by hastening the speed of diagnosis for patients with Lyme borreliosis. However, there is currently no documented evidence of any systemic and consistent social or health system change that would significantly impact speed of diagnostics over the study period.

Interestingly, the majority of the seasonal shift in cases in Norway took place preceding a period of rapid increase in case numbers and geographical range expansion (figure 1*b,d*). While we cannot document a causal relationship, the increased incidence may have been facilitated by the preceding shift in seasonality. There are many potential mechanisms by which a shift in seasonality could increase disease incidence. For example, warmer spring weather could reduce critical mortality periods for immature ticks emerging from winter diapause [23,84,89,90]. Warmer springs also could increase synchrony between larval and nymphal stages, thereby changing pathogen transmission profiles and shifting the seasonality of Lyme borreliosis risk in accordance with changes in vector stage timing [20,26,91].

This study provides quantitative evidence demonstrating seasonality changes in a vector-borne disease. Further research is needed to isolate the ecological drivers of seasonality and how phenological changes in birds, mammals and arthropods combine to impact pathogen circulation and, in turn, human disease risks.

## Data Availability

The data files and code are submitted with the manuscript as electronic supplementary material. The data are provided in the electronic supplementary material [92].
